# Eastern Ohio Dental Association

**Published:** 1871-11

**Authors:** D. B. McLain


### EASTERN OHIO DENTAL ASSOCIATION.

An adjourned meeting of a preliminary organization for the purpose of forming a dental association, was held in

Canton, on Tuesday, August 29, 1871, in J. H. Siddall's rooms.

Dr. Lyder, of Alliance, in the chair.

Dr. McLain, of New Lisbon, chairman on constitution and by-laws, reported having procured a copy of the constitution of the Northern Ohio Dental Association, which was adopted with some alterations; the name chosen being the Eastern Ohio Dental Association.

The following officers were elected for one year:

*President*—J. C. Whinery, of Salem.

*Vice President*—J. W. Lyder, of Alliance.

*Secretary*—D. B. McLain, of New Lisbon.

*Treasurer*—A. J. Douds, of Canton.

*Examining Committee*—J. H. Siddall, Canton; J. M. Porter, Massillon, and C. M. Richmond, of Garrettsville.

Convention adjourned for dinner, and were hospitably entertained by Dr. Siddall.

After dinner the following dentists were present: J. H. Siddall, L A. Hole, W. A. Porter, J. M. Porter, C. M. Richmond, J. W. Lyder, D. B. McLain, J. Craig, and A. J. Douds.

There were no members belonging to the association yet, as all must pass an examination and be recommended by the examining committee who report at next meeting, the names only of those found worthy.

Dr. J. M. Porter read a very able paper on the pathology and treatment of alveolar abscess. The subject was then discussed by nearly all present.

The subject of filling with smooth pointed instruments was next discussed, Dr. J. M. Porter having performed a clinical operation by that method in the forenoon.

The subject of specialties in dental practice was warmly discussed.

The following subjects were selected for discussion at the next meeting: Anæsthesia, The Gums and their diseases, When is Extraction Indicated?

After passing a vote of thanks to Dr. Siddall, for the use of his rooms, the society adjourned, to meet in Alliance, on last Tuesday in February next.

Alliance is the permanent place of meeting, and the meetings are to be semi-annual.

D. B. McLain, *Sec.*

